# Group a *Streptococcus* remains viable inside fibrin clots and gains access to human plasminogen for subsequent fibrinolysis and dissemination

**DOI:** 10.1128/spectrum.02607-24

**Published:** 2025-01-13

**Authors:** Henry M. Vu, Thomas E. Moran, Zhong Liang, Yun-Juan Bao, Paulina G. Carles, Jessica C. Keane, Madelyn G. Cerney, Caitlyn N. Dahnke, Ana L. Flores-Mireles, Victoria A. Ploplis, Francis J. Castellino, Shaun W. Lee

**Affiliations:** 1 Department of Biological Sciences, University of Notre Dame, Notre Dame, Indiana, USA; 2 Eck Institute for Global Health, University of Notre Dame, Notre Dame, Indiana, USA; 3 Berthiamue Institute for Precision Health, University of Notre Dame, Notre Dame, Indiana, USA; 4 W. M. Keck Center for Transgene Research, University of Notre Dame, Notre Dame, Indiana, USA; Griffith University-Gold Coast Campus, Gold Coast, Australia

**Keywords:** plasminogen, RNA-seq, fibrin, *Streptococcus pyogenes*, host-pathogen dynamics, live imaging microscopy

## Abstract

Group A *Streptococcus* (GAS) is a major human pathogen that causes several invasive diseases including necrotizing fasciitis. The host coagulation cascade initiates fibrin clots to sequester bacteria to prevent dissemination into deeper tissues. GAS, especially skin-tropic bacterial strains, utilize specific virulence factors, plasminogen binding M-protein (PAM) and streptokinase (SK), to manipulate hemostasis and activate plasminogen to cause fibrinolysis and fibrin clot escape. A major unresolved question regards the temporal dynamics of how GAS enmeshed in a fibrin clot can access plasminogen for clot dissolution and eventual dissemination. Here, we reveal through live imaging studies that GAS trapped inside a fibrin clot can remain viable in a latent state, until access to plasminogen activates fibrinolysis and dissemination. RNA-sequencing (RNA-seq) analysis shows marked changes in the wild-type (WT)-GAS transcriptome from the time bacteria were enmeshed inside the clot (4 h) to when dissemination was initiated (8 h). To gain a more fully realized model of how GAS trapped in fibrin clots can disseminate in the blood system, we utilized a novel 3D endothelial microfluidic device to demonstrate that GAS is fully capable of fibrinolysis in an endothelial environment, revealing a major underappreciated route by which GAS may cause more invasive outcomes. Our findings reveal for the first time that GAS can engage a latent, growth-suspended phase whereby physical structures such as fibrin clots that immobilize an invading pathogen allow bacteria to remain viable until sufficient access to plasminogen allows it to initiate fibrinolysis and escape into surrounding blood system and tissues.

**IMPORTANCE:**

Group A Streptococcus (GAS) is a human-specific bacterial pathogen that causes infections ranging in severity from mild to severe infections that can often be fatal. To protect the host, the innate immune system creates fibrin clots to trap bacteria and prevent deeper spread. GAS produces several factors that can initiate the dissolution of these fibrin clots to spread to deeper tissues, but we lack specific understanding of the timing of these events. Our studies demonstrate for the first time that GAS can delay their escape from fibrin clots to gain access to deeper tissues during infection, suggesting a key strategy that GAS utilize to cause more invasive disease.

## INTRODUCTION

Group A *Streptococcus* (*Streptococcus pyogenes* or GAS) is a Gram-positive bacterial pathogen responsible for a broad spectrum of infections from noninvasive clinical conditions, including pharyngitis, impetigo, scarlet fever, and cellulitis, to invasive diseases such as toxic shock syndrome and necrotizing fasciitis (1–5). Worldwide, GAS is responsible for over 1.7 million invasive infections and 500,000 deaths annually (2, 6).

An important determinant in the ability of GAS to invade deeper host tissues is the ability of the bacteria to subvert several human defenses. To bypass human physical barriers against pathogens, invasive GAS infections can penetrate via an open wound in skin and tissue into sterile environments. Skin wounds in particular create an entry point for skin-tropic GAS to allow for bacterial colonization and dissemination (6, 7). Initiation of the host coagulation cascade at the site of infection serves as a major innate mechanism to contain the bacteria, via the production of blood clots containing fibrin and other host hemostatic components (8). Fibrin clots are normally able to sequester bacteria such as GAS to limit growth and spread of bacteria into deeper tissues (9). To overcome this host response, skin-tropic strains of GAS have evolved two highly specialized virulence factors, human plasminogen (hPg)-binding M-protein (PAM) and an endogenous specific streptokinase subform (SK2b), to initiate fibrinolysis and allow for escape of GAS and dissemination into deeper tissues and blood systems (10–15). PAM, an M-protein variant specific to skin-tropic GAS strains, has an increased binding affinity for hPg, which allows for localized recruitment of hPg to the surface of GAS. Secretion of the GAS protein, SK2b, rapidly causes conversion of PAM-bound hPg to human plasmin (10, 13, 16–23), a protease that exhibits activity against a wide spectrum of substrates including fibrin, host integrins, and other cell surface proteins (15, 24–29). Previously, we demonstrated that the activation of hPg to plasmin by a skin-tropic GAS strain, AP53 (GAS_AP53_) initiates rapid and efficient fibrinolysis of fibrin clots and also triggers the compromise of cell-cell junctions through the degradation and redistribution of host integrins across cell layers (15). Therefore, activation of host hPg during GAS infection plays a critical role in the ability of GAS to not only maintain infection but to also subvert innate defenses to gain entry and access to deeper tissues and blood systems. An important, unresolved question regarding this process is the overall temporal dynamics of bacterial and cellular events that ultimately result in the ability of the bacterial pathogen to escape the enmeshed fibrin clot and disseminate into deeper tissues. In this report, we demonstrate through live imaging studies that GAS trapped inside a fibrin clot can remain viable in a latent state for several hours until access to plasminogen activates fibrinolysis and dissemination. RNA-seq analysis shows distinct changes in the wild-type (WT)-GAS transcriptome from the time bacteria were enmeshed inside the clot (4 h) to when dissemination was initiated (8 h), suggesting that large gene expression changes occur prior to plasminogen activation and fibrinolysis. To gain a more fully realized model of how GAS trapped in fibrin clots can disseminate in the blood system, we utilized a novel 3D endothelial microfluidic device to visualize the dynamics of GAS-mediated fibrinolysis in a more relevant *in situ* environment. We observed that small microcolonies of GAS that are enmeshed in fibrin clots are fully capable of fibrinolysis in a 3D endothelial environmental setting, revealing a major underappreciated route by which GAS may cause more invasive outcomes. Our findings reveal for the first time that GAS can engage a latent, growth-suspended phase whereby physical structures such as fibrin clots that immobilize an invading pathogen allow bacteria to remain viable until sufficient access to plasminogen allows it to initiate fibrinolysis and escape into surrounding blood system and tissues.

## RESULTS

### GAS trapped inside fibrin clots gains access to exogenous hPg to initiate fibrinolysis

To observe the process of GAS-mediated fibrinolysis, conditions were established to dynamically observe the process of fibrin clot dissolution by the skin-tropic GAS_AP53_ isolate that is sequestered in a fibrin clot using real-time imaging microscopy. We hypothesized that initiation of fibrinolysis by GAS inside a fibrin clot would be determined by the rate of hPg access into the fibrin clot where the bacteria are trapped. Overnight, GAS cultures were rinsed in phosphate buffered saline (PBS) before fibrinogen was added to a cell mixture at a concentration of 1 mg/mL. To convert fibrinogen to fibrin, thrombin (5 units/mL) was added to the imaging dish. This dish was set at 37°C with 5% CO_2_ for 1 h, and the samples were observed visually with microscopy to ensure the presence of bacteria and the formation of the fibrin clot within enmeshed GAS cells prior to time-lapse live imaging and addition of other components to the experiment. To enhance the resolution of clot structure imaging, fibrinogen was labeled following fibrin formation with DyLight 488 NHS-Ester, a fluorescent labeling reagent. The resulting labeled clot was allowed to incubate for 2 h after which excess reagent and bacteria not trapped in the clot were removed with two PBS washes and the samples inspected by differential interference contrast (DIC) microscopy to ensure that the fibrin clots contained enmeshed GAS bacteria. Todd-Hewitt (TH) broth and hPg (7 µg/mL) were added to the fibrin clot mixture immediately preceding the initiation of real-time imaging microscopy. Images were then obtained every 10 min for 10 h.

**Fig 1:**
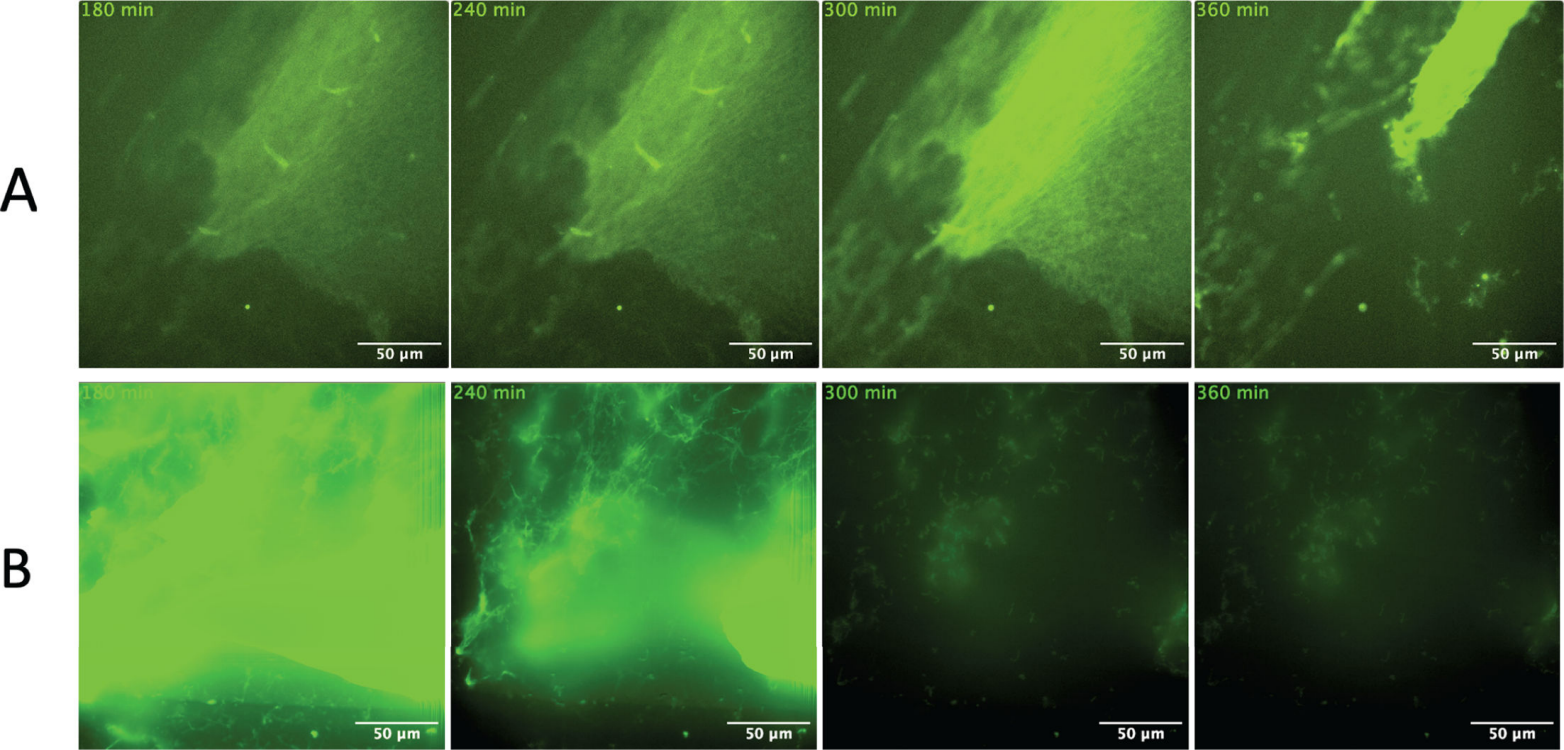
WT GAS_AP53_ triggers fibrin clot dissolution when enmeshed in fibrin clots in the presence of hPg. Fluorescently labeled fibrinogen was combined with thrombin to form fibrin clots, which entrapped GAS_AP53_ cells prior to initiation of live imaging. (**A**) hPg is added to the solution after fibrin clots are formed. (**B**) hPg is pre-incubated with GAS for 5 min prior to fibrin clot formation. Images were acquired every 10 min for 10 h for live imaging movie reconstruction. Fibrinolysis was observed at 6 h, when hPg was preincubated with GAS_AP53_ at which time rapid fibrinolysis occurs. Time-lapse images (Movies S1 and S2) are shown in panels.

Previously, we demonstrated that GAS strains expressing PAM and SK2b (e.g., the AP53 patient isolate) can recruit and activate hPg on the cell surface of the bacterium to initiate rapid fibrinolysis of mature fibrin clots, in live imaging studies (15). In this experiment, we initiated a fibrin clot that contained enmeshed GAS_AP53_ cells without hPg previously bound to GAS cell surfaces, to simulate a condition whereby GAS would not initially have sufficient access to circulating hPg. Live imaging shows that GAS trapped inside a fibrin clot that has limited access to hPg is significantly delayed in its ability to initiate fibrinolysis. From our imaging data, we first observed GAS_AP53_-mediated fibrinolysis occurring at 6 h after initiation of imaging (Fig. 1A; Movie S1). This observation demonstrates that despite sequestration of GAS within fibrin clots, bacteria can ultimately gain access to hPg circulating at physiologically relevant levels in solution in order to initiate fibrinolysis. When hPg was pre-incubated with GAS_AP53_ cells prior to clot formation, we observed dissolution of fibrin clots at earlier timepoints (~4.5 h) (Fig. 1B; Movie S2). These data show that fibrinolysis is accelerated when hPg is pre-bound to GAS_AP53_ prior to immobilization in the fibrin clot, and that access to circulating hPg by GAS inside the fibrin clot determines the overall timing of the fibrinolytic event.

### Escape from fibrin clots by GAS requires SK for hPg activation

**Fig 2:**
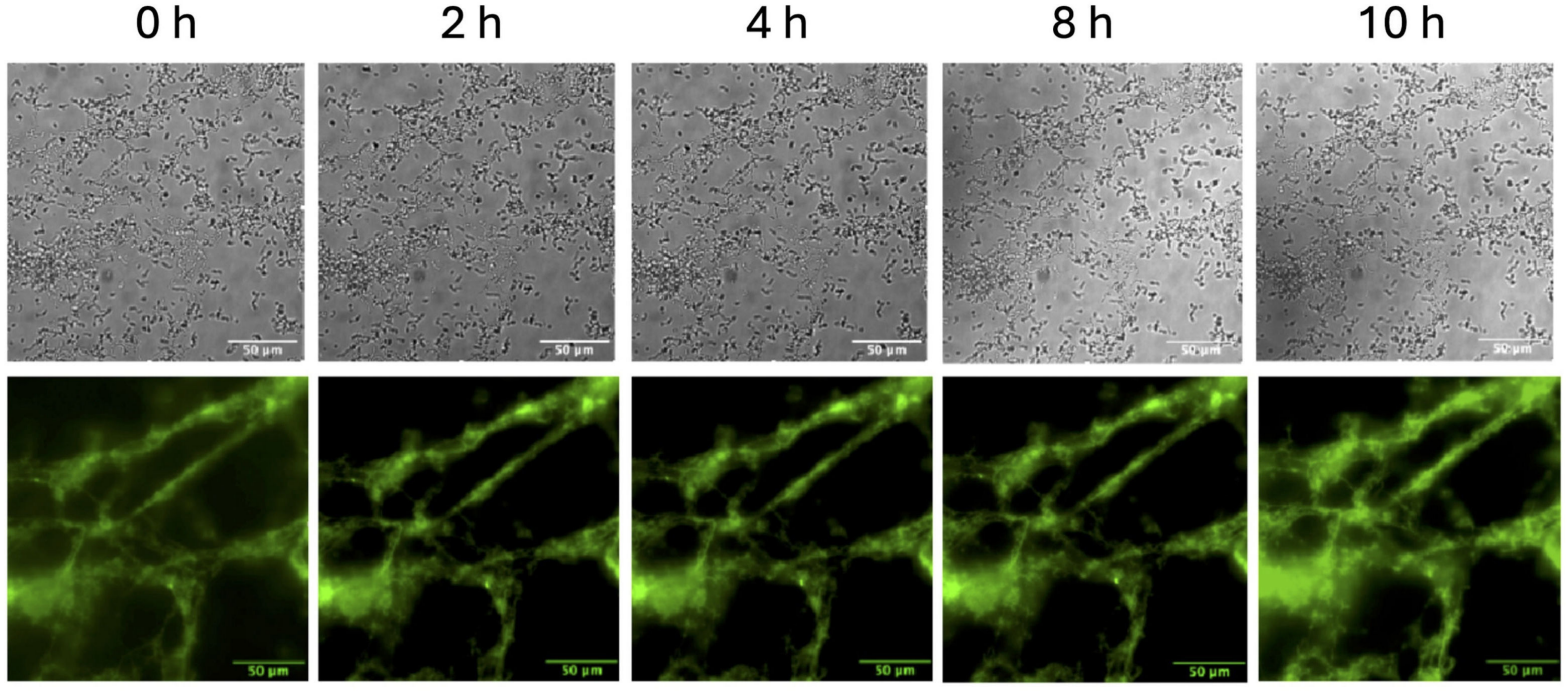
SK is required for hPg activation and fibrinolysis by GAS. The GAS_AP53_::ΔSK isogenic mutant remains enmeshed inside a fibrin clot over the course of 10 h in the presence of hPg and TH media. DIC images (top panels) show immobilized GAS_AP53_ bacteria without growth or movement over the course of 10 h. Fluorescently labeled fibrin was imaged along with DIC capture of the GAS_AP53::_ΔSK inside the fibrin clot (bottom panels), confirming that there was no fibrinolytic activity observed over the course of 10 h. Fibrin structures retained their original structure throughout the 10 h. See also Movies S3 and S4.

We next sought to confirm the critical function of the GAS-secreted protein, SK, in the ability of GAS to initiate fibrinolysis via hPg activation and conversion to plasmin. Using identical conditions as above to observe GAS escape from fibrin clots, we observed no dissemination of GAS enmeshed in fibrin clots in our GAS_AP53_/ΔSK isogenic mutant, over the course of 10 h (Fig. 2; Movies S3 and S4). Fluorescently labeled fibrin was imaged along with DIC capture of the GAS_AP53_/ΔSK mutant inside the fibrin clot, confirming that fibrinolytic activity was absent over the course of 10 h and that the fibrin retained original structure over 10 h with bacteria intertwined inside the fibrin clot in DIC images. This finding importantly confirms that the fibrinolytic events that were previously recorded using the GAS_AP53_ strain require SK-mediated activation of hPg to escape the clot.

### GAS remains viable while encased in fibrin clots in solution

**Fig 3:**
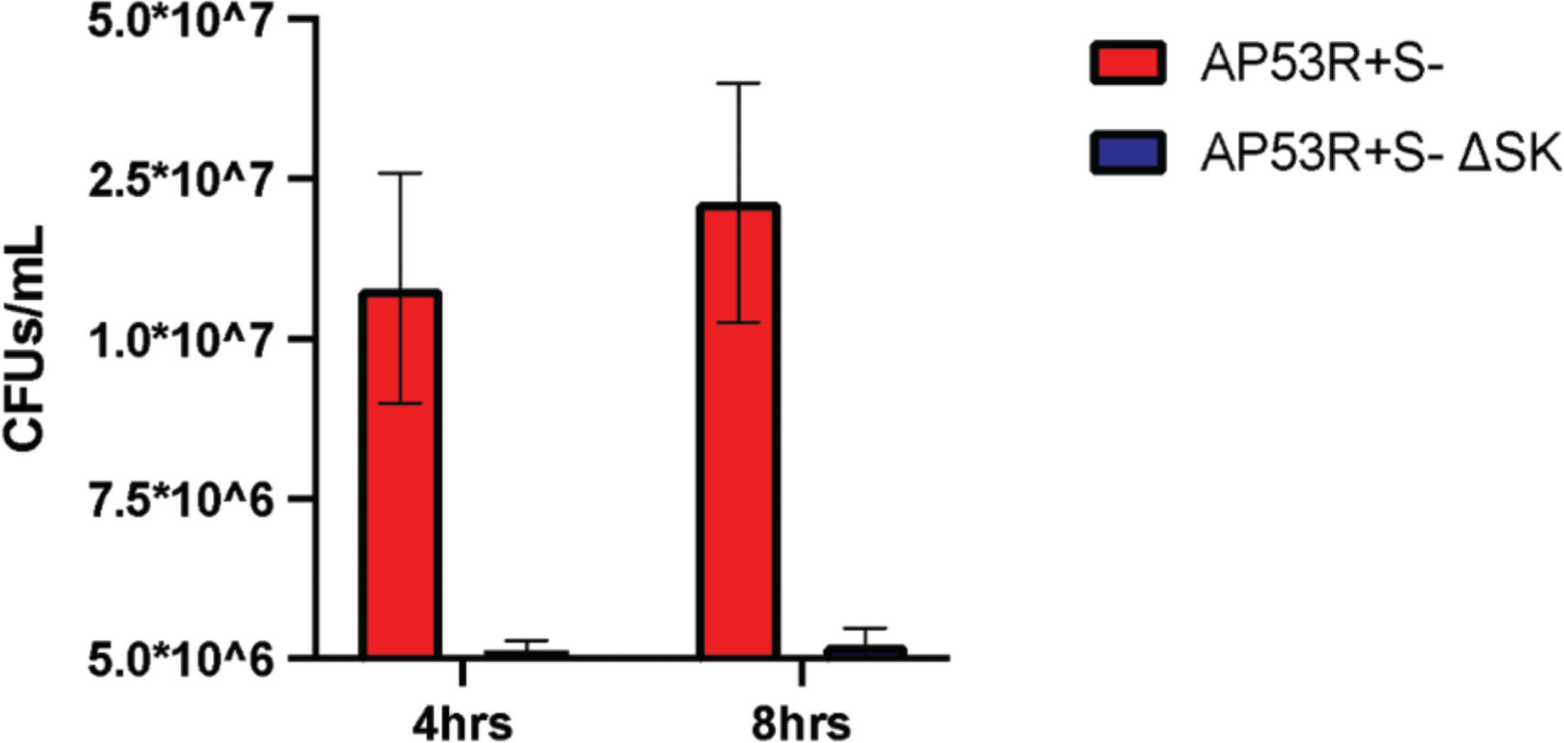
WT-GAS_AP53_ and GAS_AP53/_ΔSK remain viable while encased in fibrin clots. GAS_AP53_ isolated from mechanically disrupted fibrin clots after incubation for 10 h was plated on TH plates to quantify growth of GAS at time points and treatment conditions. Bacterial CFU counts were quantified and performed in triplicate for significance. The CFUs of WT-GAS_AP53_ are in red, and those of GAS_AP53_::ΔSK are shown in blue. Error bars are indicated in the graph of triplicate CFU sampling (*P* < 0.0001). The time-lapse movie shows that the bacteria displayed no visible sign of growth or division during the 10 h time course (Movie S1).

Live imaging data showed that during the period when GAS_AP53_ cells are sequestered inside the fibrin clot, there was no significant growth of GAS, indicating that rapid growth of the bacterium is only observed after fibrinolysis occurs and the bacterium subsequently escapes from the fibrin clot (Fig. S1; Movie S5). Therefore, we wanted to determine if GAS cells that are trapped inside the clot without access to hPg would continue to remain viable and metabolically active in some form. Fibrin clots with GAS_AP53_ enmeshed were formed following the same procedure used for GAS strains (GAS_AP53_ and GAS_AP53_/ΔSK). These GAS-embedded clots were incubated for 4 h and 8 h. Following incubation, the clot was mechanically disrupted in TH solution prior to plating on TH agar to quantify the growth of GAS. Quantification of bacteria revealed that GAS_AP53_ remained viable while enmeshed in the clot without appreciable growth from the period of 4–8 h when enmeshed in the clot structure (Fig. 3). For the WT-GAS_AP53_ strain, an average of ~1 × 107 CFUs were recovered from the collected fibrin clots at 4 and 8 h, indicating that there was not significant growth over the course of 8 h, even though most of the bacteria remained viable inside the fibrin clot. A similar trend occurred with the GAS_AP53_/ΔSK strain, with an average of ~5 × 106 CFUs recovered from the fibrin clot. The bacteria displayed no visible sign of growth or division during the observed time course even though our experiment was performed in TH broth, which is permissible for GAS growth. GAS growth in permissible TH broth is typically observed to have a log growth phase in the period of 4–8h following initial inoculum. However, our data show that when the bacteria are enmeshed in the clot, in the absence of fibrinolysis, the GAS bacteria remain in a static phase with very little growth overall. The variance in starting inoculum for both conditions is largely due to the fact that even though we initiated the experiment with the same number of GAS bacteria, the process whereby we initiate fibrin clots to trap them in the clot, followed by rinse steps to remove untrapped bacteria, introduces variations in the overall number of bacteria measured in this study. However, our data confirm the observations made through live imaging, specifically that from 4 to 8h of GAS enmeshed in the fibrin clot, there is very little overall growth observed. Significantly, our findings demonstrate that when GAS cells are trapped within a fibrin clot, they enter a state in which growth and division are suspended while nonetheless remaining viable. This is the first observation that GAS cells can remain viable for up to 8 h while not engaging in growth in the presence of nutrient-rich conditions.

### GAS cells remain transcriptionally active while embedded in fibrin clots in solution

We next performed RNA-seq analysis of WT-GAS_AP53_ and the isogenic GAS_AP53_/ΔSK mutant to better understand SK-dependent transcriptional changes that occurred during the latent phase of the bacteria trapped inside the fibrin clot (Data S1).

**Fig 4:**
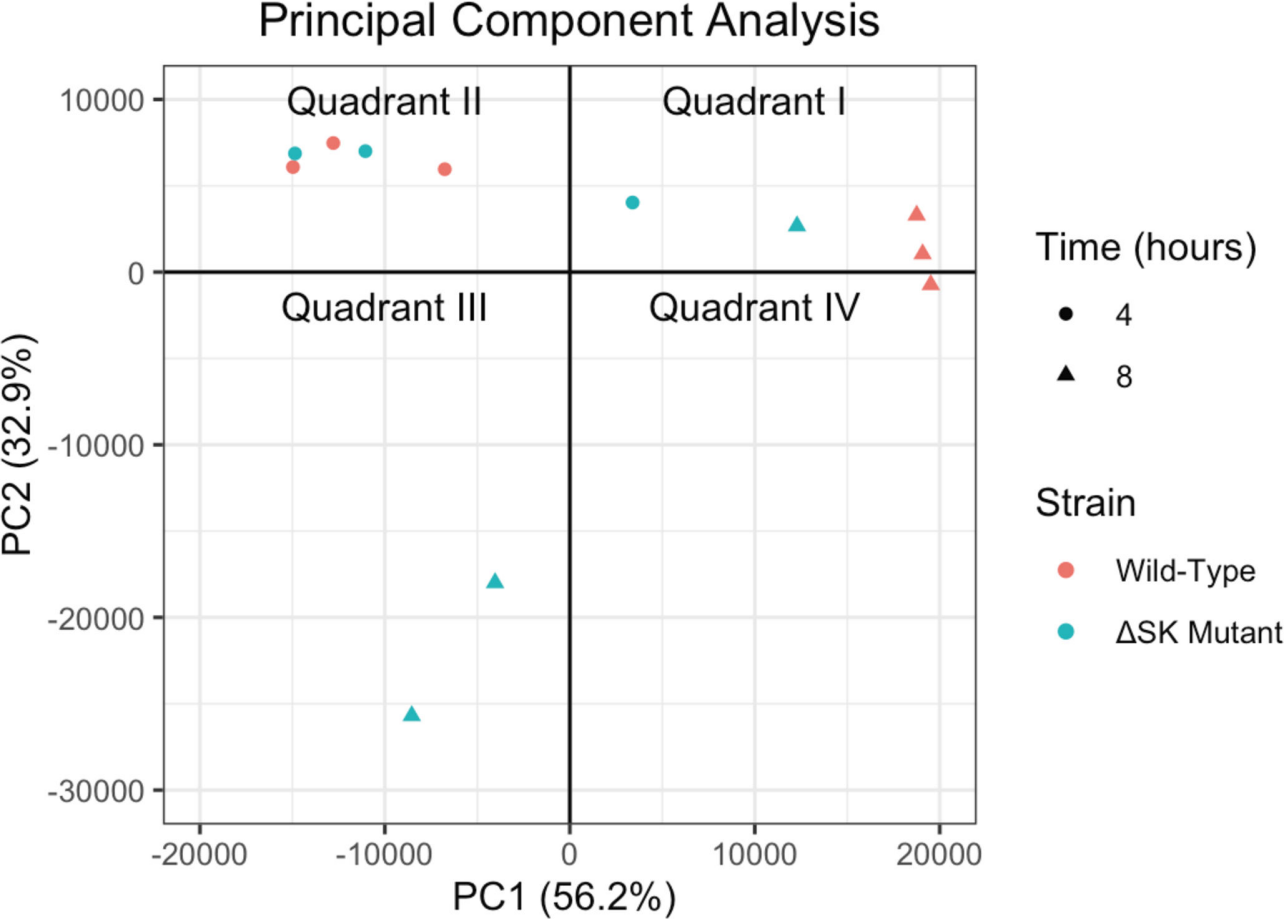
PCA of WT GAS_AP53_ and GAS_AP53_/ΔSK transcriptomes at 4 and 8 h. Differential expression values were acquired through RNA-seq using an Illumina MiSeq platform. WT- GAS_AP53_ and mutant GAS_AP53_::ΔSK cultures were grown overnight and entrapped in the clot. After clot formation, hPg (7 µg/mL) was added. The clot was then incubated at 37°C at times of 4 and 8 h. Three replicates of each GAS_AP53_ strain were processed for RNA-seq. Data for WT-GAS_AP53_ are shown in blue, and data for GAS_AP53_::ΔSK strains are in red. Transcriptomes of the 4-h WT-GAS_AP53_ and GAS_AP53_/ΔSK mutant strains are similar based on clustering pattern indicated in quadrant II of the PCA graph. Transcriptomic profiles of WT-GAS_AP53_ in the fibrin clot at 8 h also shown clustering as in quadrants I and IV.

**Fig 5:**
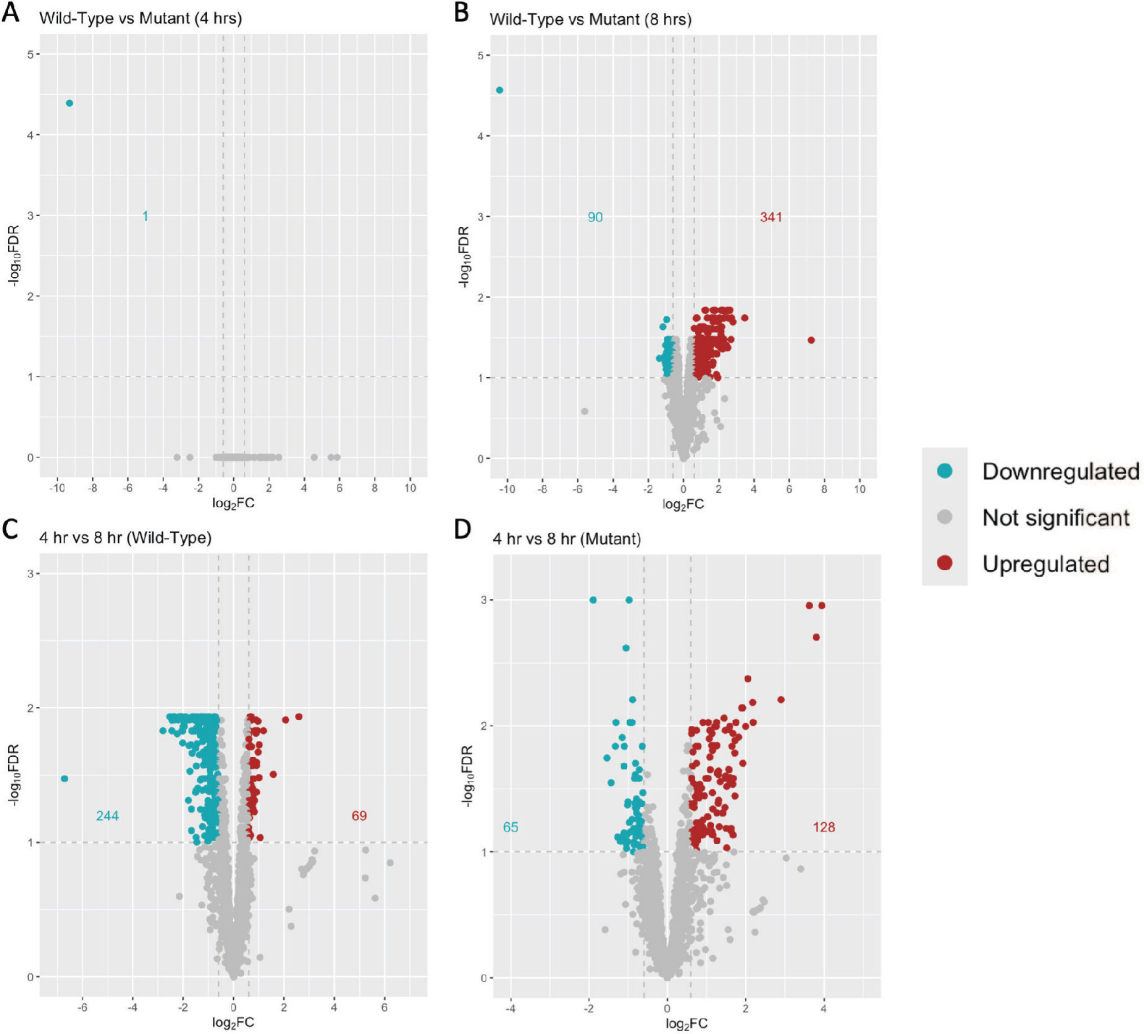
Transcriptional analysis of differential gene expression of GAS trapped in fibrin clots. RNA-seq analyses of WT-GAS_AP53_and GAS_AP53_::ΔSK were compared along with 4- and 8-h time points of each GAS strain in a trapped clot. The graphs show (**A**) transcriptional differences at 4 h between GAS_AP53_ and GAS_AP53_::ΔSK, (**B**) transcriptional differences at 8 h between GAS_AP53_ and GAS_AP53_::ΔSK, (**C**) transcriptional differences between 4 and 8 h in WT-GAS_AP53_, and (**D**) transcriptional differences between 4 and 8 h in GAS_AP53_::ΔSK. The ordinate axis shows the log_2_ fold changes in transcript. Upregulated genes are identified in red, downregulated genes are identified in blue, and gene expression changes that exceed a FDR threshold of >0.1 and a log_2_ fold change threshold of < Δ0.6 (Δ1.5 fold change) are in gray. The number of genes meeting these thresholds is denoted on the graph in red text for upregulated genes and in blue text for downregulated genes. A complete profile of all gene expression changes is listed in supplemental data (Data S1).

Principal component analysis (PCA) of the data obtained from RNA sequencing revealed that the transcriptomes of the 4-h WT-GAS_AP53_ and GAS_AP53_/ΔSK mutant strains maintained remarkable similarities based on their clustering pattern in quadrant II of the PCA graph (Fig. 4). Transcriptomic profiles of WT-GAS_AP53_ in the fibrin clot at 8 h also clustered closely together (quadrants I and IV), while the 8-h GAS_AP53_/ΔSK transcriptomes diverged greatest along the PC2 axis. However, using a threshold false discovery rate (FDR) of <0.1, we found that the differential expression of genes between our WT-GAS_AP53_ and GAS_AP53_/ΔSK strains progressed from one gene at 4 h (encoding SK) to 702 genes at 8 h, with 431 genes showing a log fold change of >1.5 or <−1.5 (Fig. 5A and B). These findings show that there arose pronounced variation in 22.2% of the transcriptome between the WT and mutant strains from 4 to 8 h, suggesting that the SK gene in GAS may play an important role in directing the GAS transcriptional response to fibrin entrapment.

### GAS decreases general transcriptional activity and upregulates virulence-associated genes while trapped in a fibrin matrix

To further understand the general GAS transcriptional response to fibrin trapping, we compared the overall changes in gene expression of WT-GAS_AP53_ between 4 and 8 h while enmeshed in the fibrin clot. According to our live imaging data reported earlier, by 8 h, this GAS strain has converted hPg to plasmin to initiate fibrinolysis and escape from the fibrin clot matrix, while remaining undivided (Fig. 1 and 3). Comparison of the transcripts at 4 and 8 h showed that differential expression was detected in 313 genes or 16.1% of the transcriptome, with 244 genes downregulated and 69 genes upregulated using our ± 1.5 fold change threshold.

**Fig 6:**
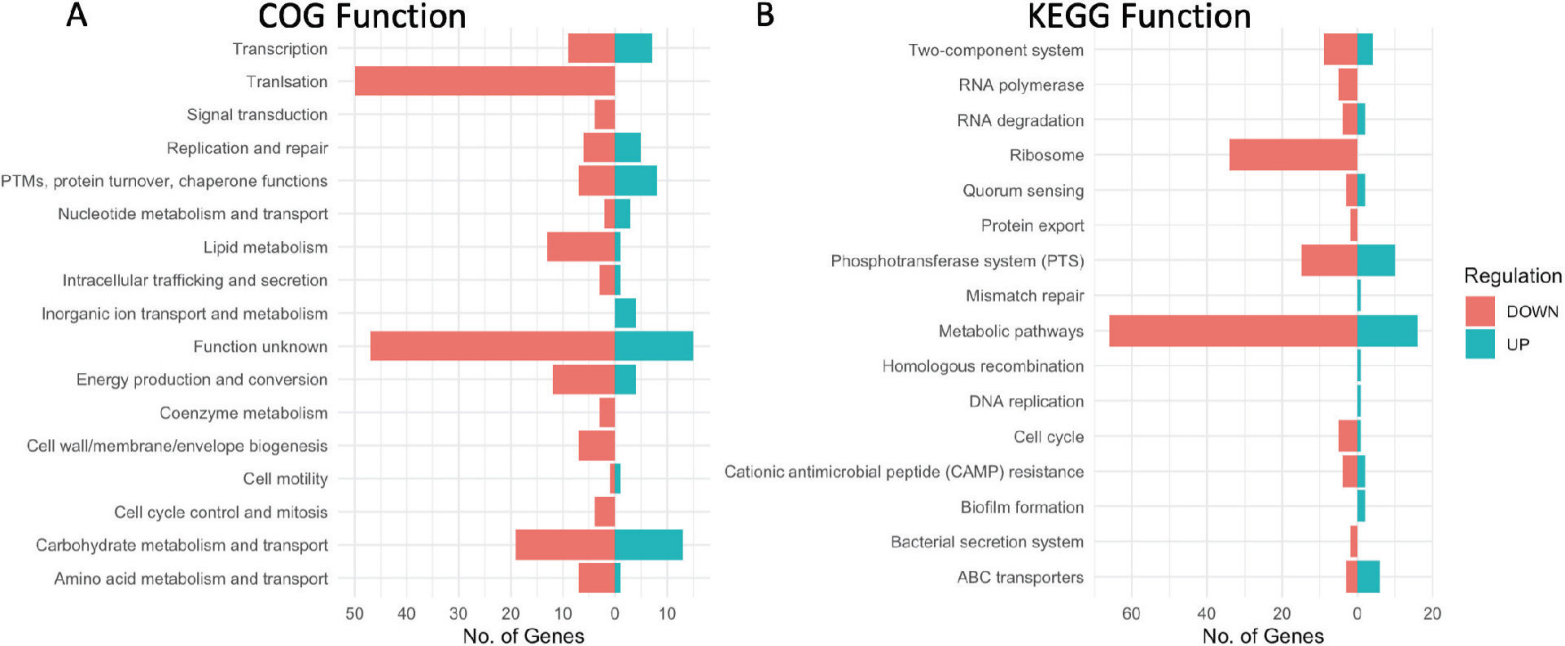
Profile of differentially expressed genes for fibrin-trapped WT-GAS_AP53_ at 4 and 8 h. Numbers of genes that showed differential expression values > Δ0.6 log_2_ fold change (Δ1.5 fold change) between 4 and 8 h in WT-GAS_AP53_ entrapped in fibrin. (**A**) COG categories were assigned to 149 genes of 313 differentially expressed genes. (**B**) KEGG categories were assigned to 169 genes of 313 differentially expressed genes. Some genes were assigned more than one COG/KEGG category as resulting proteins are involved in multiple pathways or serve more than one function. The FDR threshold <0.1 *x*-axis in graphs shows number of genes. Upregulated genes are identified in blue; downregulated genes are identified in red.

We annotated our AP53 GAS genome using the Cluster of Orthologous Genes (COG) and the Kyoto Encyclopedia of Genes and Genome (KEGG) databases. Using these databases, we assigned COG classifications to 1,576 of the 1,941 sequences (81% coverage), while KEGG classifications were assigned to 738 (38%). We reported the number of genes assigned to each COG and KEGG category that were upregulated or downregulated using an FDR threshold of 0.1 and a differential expression value >1.5 or <−1.5 (Fig. 6). Overall, these analyses revealed that differential expression decreased overall between 4 and 8 h while bacteria are enmeshed in the fibrin clot. Decreased gene expression occurred largely in metabolic pathways, as well as in genes involved in protein translation.

KEGG analysis identified 66 metabolic pathway genes to have decreased differential expression in the WT-AP53 strain between 4 and 8 h (Fig. 6B). Among these metabolic pathways that were downregulated, we found multiple genes involved in fatty acid biosynthesis, glycolysis, ATP-synthesis (F-type ATPase), and gene-encoding enzymes for fructose and mannose metabolism. Of the upregulated metabolic genes, we identified several that were involved in galactose and starch/sucrose metabolism. Interestingly, the transcriptional repressor DeoR was upregulated, which inhibits the transcription of ribonucleoside and deoxyribonucleoside catabolizing enzymes in *Escherichia coli* (30), although a *deo* operon has not been characterized in this GAS strain. Overall, the transcriptional state of GAS as it is enmeshed in clots just prior to escape is indicative of an overall repressed metabolic state, indicative of latency.

In contrast to the general downregulation of metabolic genes, transcript analysis showed the notable increase in expression for several virulence factor genes between 4 and 8 h timepoints. Between 4 and 8 h, WT-GAS_AP53_ had increased transcription of 10 notable virulence factors that included genes *sagABC*, *speB*, *spi*, *speK*, *spd*, *saPIn2*, *ndoS*, and *cfa* (Table 1). The product of *sagA* expression is a precursor peptide for streptolysin S (SLS), a potent hemolytic and cytolytic exotoxin which requires several genes for complete biosynthesis that are found in the *sag* operon (31–33). The gene *cfa* encodes the pore-forming CAMP factor which has hemolytic activity along with inhibitory effects on phagocytosis (34, 35). The *speB* gene encodes a cysteine protease that is required to induce necrotizing *fasciitis* in mouse infection models (36). This promiscuous protease has been shown to inactivate a host of immune proteins including cytokines, chemokines, complement components, extracellular matrix, and immunoglobulins (37). *Spd* encodes extracellular nucleases that have been shown to allow GAS escape from neutrophil extracellular traps (38). We note here, however, that the GAS_AP53_ strain we used for these studies is a human skin-tropic isolate in which the CovRS global virulence regulator has been naturally inactivated. This may indicate that these strains maintain production of virulence factors, especially those involved in degradation of host components (DNA, protein, and erythrocytes) in order to prepare for subversion of host defenses once they escape from the enmeshed clot into the general host blood system and tissues.

**TABLE 1:** Virulence factors with differential expression during fibrin entrapment

Gene	Log (fold change)			Description	Citation
	4 h v 8 h		WT v ΔSK		
	WT	ΔSK	8 h		
*sagA*	1.58	3.81	2.16	SLS biosynthesis protein A	(31)
*sagB*	0.75	1.09		SLS biosynthesis protein B	(31)
*sagC*	0.66	1.14		SLS biosynthesis protein C	(31)
*sagD*		1.26	0.58	SLS biosynthesis protein D	(31)
*sagE*		1.08	0.64	SLS self-immunity protein	(39)
*sagF*		1.20	0.83	SLS biosynthesis protein (membrane associated)	(40)
*sagG*		1.45	0.90	Export ABC transporter ATP-binding protein; SLS export protein	(31, 39)
*sagH*		1.48	0.63	SLS export transmembrane permease	(31, 39)
*sagI*		1.57	0.77	SLS export transmembrane permease	(31, 39)
*sclA*	−0.61			Collagen-like surface protein	(41)
*emm49*	−0.45	−0.72		Anti-phagocytic M protein	(42)
*slaA*		1.72	1.75	Streptococcal phospholipase A2	(43)
*prtF2/fbaB*	−1.33		1.43	Fibronectin-binding protein	(44)
*sodA*			0.95	Superoxide dismutase	(45, 46)
*hylP*	−1.25	−0.86		Hyaluronate lyase	(47)
*spd*	1.06	2.90	1.61	Streptodornase B	(38)
*spd3*		1.06	0.84	Streptococcal extracellular nuclease 3	(48)
*mcra*		0.99	0.89	67-kDa myosin cross-reactive antigen FAD Enzyme	(49)
*shr*			−0.61	Streptococcal cell surface hemoprotein receptor	(50)
*cfa*	0.52			CAMP factor	(51)
*speB*	1.18			Cysteine proteinase	(37, 52)
*spi*	1.01	0.76		SpeB inhibitor	(53)
*speK*	0.61		−0.55	Superantigen	(54)
*SaPln2*	0.48	0.45		*Staphylococus aureus* pathogenicity island, SaPI2-like genetic element	(55)
*sse*			−0.29	Secreted esterase	(56)
*ndoS*	0.87	0.57		endo-β-N-acetylglucosaminidase, Ig-G degrading enzyme	(57)
*pepO*	−1.11			Neutral endopeptidase O, plasminogen-binding and fibronectin-binding	(58)

In contrast to the WT-GAS_AP53_ transcriptome, the GAS_AP53_/ΔSK mutant responded to the fibrin trapping with a collective upregulation in differentially expressed genes at 8 h compared to 4 h. Notably, GAS_AP53_/ΔSK cells cannot initiate fibrinolysis. Thus, while WT- GAS_AP53_ is escaping the fibrin clot, GAS_AP53_/ΔSK cells remain trapped at 8 h. Of the 193 differentially expressed genes identified (9.9% of transcriptome), 128 genes were upregulated, while 65 genes were downregulated (Fig. 5D).

**Fig 7:**
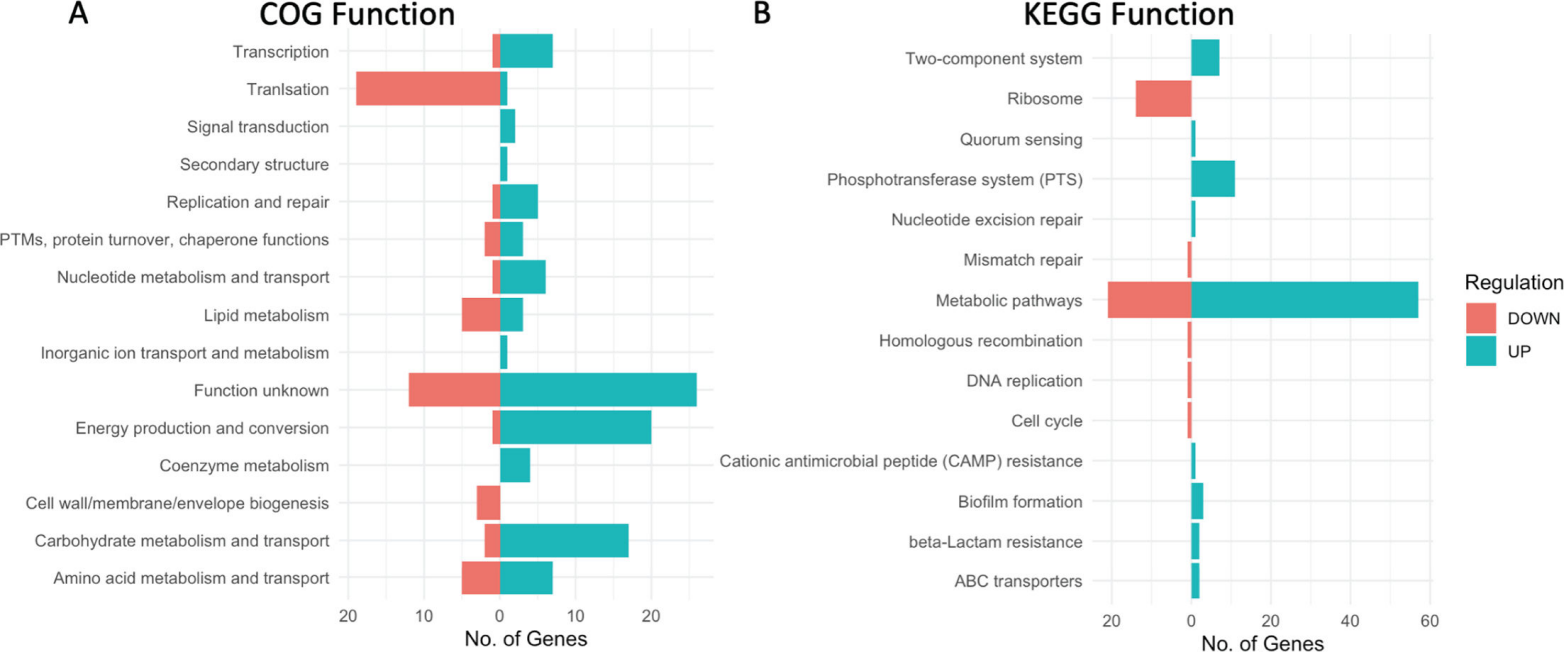
Profiles of differentially expressed genes for fibrin-entrapped GAS_AP53_::ΔSK at 4 and 8 h. Genes showing differential expression values > Δ0.6 log_2_ fold change (Δ1.5 fold change) between 4 and 8 h during GAS_AP53_::ΔSK entrapped in fibrin. (**A**) COG categories were assigned to 159 genes of 193 differentially expressed genes. (**B**) KEGG categories were assigned to 116 genes of 193 differentially expressed genes. Some genes were assigned more than one COG/KEGG category as resulting proteins are involved in multiple pathways or serve more than one function. The FDR threshold <0.1 *x*-axis in graphs shows number of genes. Upregulated genes are identified in blue; downregulated genes are identified in red.

COG and KEGG assigned functions indicate that metabolic pathways were primarily upregulated (Fig. 7). Interestingly, ribosomal/translational genes were primarily downregulated, although to a lesser degree than what we observed in the WT-GAS_AP53_ cells. For KEGG annotations, 57 upregulated genes were involved in various metabolic pathways (Fig. 7B). Among these genes were several involved in histidine metabolism, as well as metabolism of pyruvate and purines. It is clear from this analysis that the inactivation of SK has a significant effect on the transcriptional regulation by GAS while remaining in fibrin clots. Without the ability to produce SK, GAS was found to be generally more metabolically active and expressed several virulence factors at a higher rate (Table 1). Our results indicate that the expression of the SK gene is important in orchestrating the transcriptional response of GAS_AP53_, influencing the timing of metabolic and other regulatory processes.

The genes showed differential expression values > Δ1.5 fold change between 4 and 8 h during GAS_AP53_/ΔSK entrapped in fibrin. (i) COG categories were assigned to 159 genes of 193 differentially expressed genes. (ii) KEGG categories were assigned to 116 genes of 193 differentially expressed genes. Some genes were assigned more than one COG/KEGG category as resulting proteins are involved in multiple pathways or serve more than one function. The FDR threshold <0.1 *x*-axis in graphs shows number of genes. Upregulated genes are identified in blue; downregulated genes are identified in red.

Taken together, the data obtained from our transcriptome analysis demonstrate that dynamic transcription states by GAS are evident while the bacteria remain trapped in a fibrin clot. Our findings show for the first time that GAS can engage a latent, growth-suspended phase whereby physical structures such as fibrin clots that immobilize an invading pathogen allow bacteria to remain viable and transcriptionally active for an extended time during host infection. GAS that is trapped in a fibrin clot will therefore enter a state in which the bacteria suspend growth, but remain viable, until sufficient access to hPg allows it to initiate fibrinolysis and escape into surrounding tissues.

### Visualization of GAS-mediated fibrinolysis *in situ* using a 3D-engineered endothelial environment

**Fig 8:**
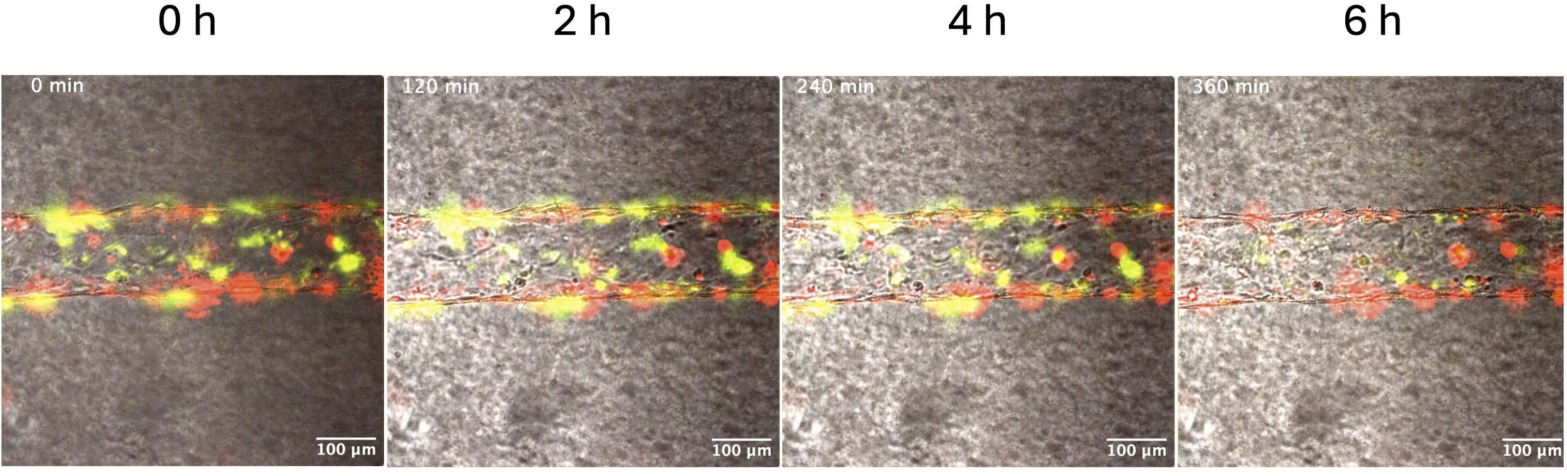
GAS_AP53_ mCherry infection of an EMV device containing fibrin clots and HUVECs. Under the infection conditions where GAS is not trapped in fibrin and hPg is added simultaneously (175 µg/mL), fibrin (labeled green) undergoes rapid fibrinolysis in under 6 h. GAS_AP53_ expressing mCherry is indicated in red. See also Movie S6.

**Fig 9:**
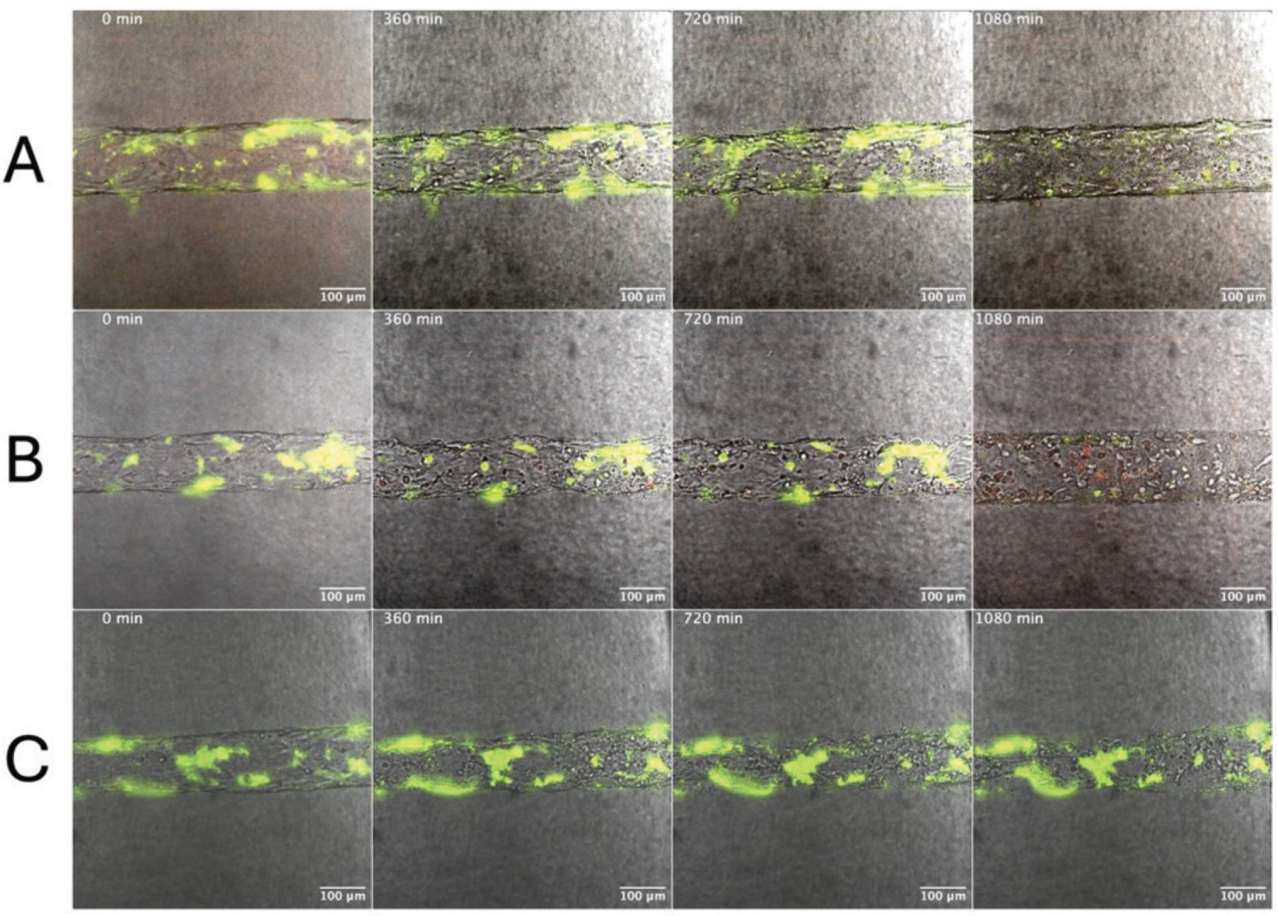
Fibrin-entrapped GAS_AP53_ mCherry GAS infection of an EMV device containing fibrin clots and HUVECs. Under infection conditions where GAS_AP53_ is trapped in fibrin and hPg is added simultaneously (175 µg/mL), fibrin (fluorescently labeled in green) undergoes fibrinolysis at ~18 h after being introduced into the sytem (top row). Under infection conditions where hPg (175 µg/mL) is pre-incubated with GAS_AP53_ prior to fibrin entrapment, fibrin clots undergo fibrinolysis in the same time period (18 h) in the EMV device (middle row). Fibrinolysis with GAS_AP53_::ΔSK enmeshed in clots does not occur after 24 h of imaging, even as the endothelial structure begins to eventually degrade. GAS expressing mCherry is indicated in red. See also Movies S7 and S8.

To gain a better understanding of how GAS_AP53_ trapped in fibrin clots would initiate dissolution and subsequent dissemination in a physiologically relevant setting, we developed a novel live imaging technique of visualizing the process of GAS-mediated fibrinolysis in a 3D endothelial setting akin to a large human blood vessel. We fabricated an engineered microvessel (EMV) device (39) and adapted the device for live imaging and visualization of sustained host–pathogen interactions in a more appropriate *in situ* environment. The EMVs we used in this experiment contained human umbilical vein endothelial cells (HUVECs) growing on a collagen type I hydrogel substrate. The EMVs were subsequently optimized for the incorporation of fibrinogen GAS_AP53_, hPg, and thrombin to observe fibrinolysis in an *in situ* 3D endothelial environment. In the first experiment, a fibrin clot was incorporated into the 3D device, followed by the addition of GAS_AP53_ and hPg. Under these conditions, in which GAS_AP53_ and hPg are freely circulating in the endothelial environment, we observed fibrinolysis at 6 h post-addition (Fig. 8; Movie S6). We next performed an experiment in which GAS_AP53_ are first enmeshed in fibrin clots, prior to addition into the EMV. To accomplish this, we incubated fibrinogen, thrombin, and GAS for 1 h to allow the GAS–fibrin complex to form before we added the aggregate to the EMV device. In one set of experiments, hPg was added to the device immediately before imaging. We observed that under these conditions, fibrinolysis by GAS_AP53_ can be detected in the EMV after an incubation period of ~18 h (Fig. 9A; Movie S7). This further supports our previous data showing that GAS_AP53_ growth can be substantially suspended while they are enmeshed in fibrin clots but that the GAS_AP53_ cells remain viable and can gain access to circulating hPg in the endothelial microvessel environment to eventually cause fibrinolysis and escape the clot complex. We also performed experiments similar to those described earlier wherein we observed that pre-incubating hPg with GAS_AP53_ led to an earlier onset of fibrinolysis in our *in vitro* setting. Using our EMV system, when GAS_AP53_ was pre-incubated with hPg prior to clot formation, we observed that fibrinolysis mediated by GAS_AP53_ inside the 3-D endothelium was still observed at 18 h (Fig. 8B; Movie S8). Similar to prior studies, the GAS_AP53_/ΔSK mutant was unable to initiate fibrinolysis and escape into the 3D endothelial environment over a time period of 24 h (Fig. 8C). This finding provides an important temporal distinction between our *in vitro* and *in situ* conditions and emphasizes the need for physiologically simulated conditions such as our EMV device to more closely model the events in fibrinolysis and dissemination observed during human infection by GAS. Our data provide evidence that during bacterial entry into the blood systems, any GAS bacteria that are trapped in the fibrin clot that is circulating in the blood can undergo an extensive period of latency (over 12 h) in which they can remain viable and eventually escape to cause further dissemination once they gain access to circulating hPg.

## DISCUSSION

In this study, we provide the first real-time live image capture of pathogenic GAS_AP53_ trapped inside a fibrin clot over the course of 24 h. Our data reveal the striking finding that GAS_AP53_ can enter a latent state whereby growth and bacterial division are greatly attenuated. However, the bacteria inside the fibrin clot remain transcriptionally active during the latency phase. It has not previously been reported that GAS can enter a latent phase inside fibrin clots. In our live imaging conditions, we incubated the fibrin-enmeshed GAS_AP53_ in the presence of TH broth, thereby providing nutrient-rich conditions for access by GAS trapped in the clot. As a result, we speculated that GAS would have the ability to grow inside the trapped clot over time and that we would observe a phenotype where the bacterial growth would allow eventual escape from the fibrin structure. However, our live imaging reveals that GAS cells trapped inside the clot in the absence of hPg do not have the ability to escape the fibrin clot over the course of infection. Thus, hPg activation remains a critical event by which GAS_AP53_ can initiate fibrinolysis to escape from the enmeshed clots to disseminate into deep tissue sites. Our imaging demonstrates that when hPg is absent, or when SK is inactivated in GAS, fibrinolysis does not occur over the time course observed.

We also reveal with our live imaging studies that hPg added exogenously to the enmeshed fibrin clot will eventually diffuse into areas where GAS and SK can activate hPg, thereby initiating rapid fibrinolysis. Previous reports have shown that permeability of fibrin constructs depends on the concentration of fibrinogen and thrombin used to make the construct. Even at high concentrations of fibrinogen (15 mg/mL), some amount of high-molecular-weight (70,000 kDa) molecules can permeate fibrin structures (40). We observed fibrinolysis by GAS_AP53_ at 6 h post-incubation, at which time fibrinolysis happened rapidly, presumably by amplified human plasmin generation. The lack of any movement by GAS_AP53_/ΔSK when enmeshed in the clot with hPg demonstrated that in general, our protocol resulted in GAS bacteria that were generally rendered immobile and incapable of undergoing binary fission. Although we had created fibrin clot structures with enmeshed bacteria, our studies at present cannot distinguish between GAS that is immobilized in the center matrix of the clot vs those enmeshed closer to the exterior of the clot. We have designed the experiments to ensure that any GAS that is not immobilized in the clot is to be removed prior to the start of the live imaging experiment.

Together, these data confirm that GAS trapped inside fibrin clots can gain access to sufficient hPg in solution for SK-dependent activation and initiation of fibrinolysis. In our experiments, we were careful not to exceed the typical concentration of hPg used that would be estimated to mimic a physiologically relevant level of circulating hPg. Our results therefore likely reflect conditions appropriate for a given *in vivo* infection setting, in which skin-tropic GAS can initiate an infection at a site of skin breach and persist in a viable state, especially as innate defenses initiate hemostasis to contain the spread of bacteria and bleeding.

To better understand the state of viable GAS trapped inside a fibrin clot without access to hPg, we performed RNA-seq analysis of WT- GAS_AP53_ and the isogenic SK-inactivated mutant to understand both global and SK-dependent transcriptional changes during GAS–fibrin entrapment. We hypothesized that genes involved in transcription would continue to be active. Indeed, a survey of transcriptional changes indicated that many of these genes (e.g*.*, rRNA subunit genes, tRNA, and ribosomal subunit genes) were sustained over the course of incubation albeit at reduced levels (Data S1). RNA-seq analysis using KEGG classifications to the transcriptome revealed overall downregulation of many cellular pathways in WT-GAS_AP53_ between 4 and 8 h. These results suggest GAS enters a latency state which involves transcriptional processes beyond cell division and may be a part of a pathogenic strategy that is not fully understood at this time. By contrast, transcriptional analysis of GAS_AP53_/ΔSK reveals a distinguishable response to fibrin entrapment characterized by global upregulation of genes. This polarized response, affecting over one-fifth of the transcriptome, highlights the potential significance of SK in regulating GAS–fibrin clot interactions. These transcriptional distinctions are consistent with the dramatic phenotypic outcomes that we observe in live imaging during GAS infection. Given thsese findings, however, we cannot provide conclusive evidence at this time that the presence of SK has a *direct* role in affecting transcription based on our findings. Indeed the possibility that limited fibrin degradation by WT bacteria may simply improve movement and ability to acquire nutrients over SK remains a valid reason for the data that we observe in our imaging. The intriguing speculation that SK may play a role in sensing the process of fibrinolysis in GAS will need further study. Previous studies by Sun et al. (12) showed that inhibiting SK via chemical inhibitor did not affect the general growth of GAS but that the chemical had the significant ability to decrease GAS infection mortality (dissemination) in their mouse model. Furthermore, the inhibition of SK in their studies was shown to also alter a network of virulence traits, suggesting that SK may somehow be linked to the control of other virulence traits. Therefore, it is interesting to hypothesize that the production and activation of SK on the surface of GAS highly influences downstream processes, based on both our findings and previous work. Future studies are warranted to gain more insights into the possible connection between SK and the regulation of GAS virulence traits.

We are currently pursuing studies to identify and analyze the profile of genes that are regulated in an SK-dependent manner and to better understand possible mechanisms of GAS latency and reactivation, especially in the context of a skin-based infection that involves the host activation of hemostasis and innate defenses.

The observation of transcriptional downregulation of nutrient utilization genes observed in GAS_AP53_ from 4 to 8 h in our studies may indicate the overall shift that GAS undergoes as it escapes the enmeshed fibrin clot. Initially, at 4 h, metabolic gene expression may be maintained overall in order that GAS trapped in the fibrin clot can continue to remain viable and in a latent state. By comparing this to 8 h, a timepoint at which GAS has escaped the fibrin clot, the overall downregulation of metabolic genes that we observed compared to the 4-h time likely indicate that the bacteria can respond by dedicating less energy and resources into obtaining nutrients, as it has now full access to the surrounding nutrient-rich environment.

Finally, we developed and utilized an *in situ*-based 3D endothelial microvessel environment to gain additional insights into the dynamics of GAS-mediated fibrinolysis and how this setting would compare to our *in vitro* fibrinolysis experiments. Strikingly, we found that GAS_AP53_ cells enmeshed in a fibrin clot that is inserted into the 3D endothelial environment were still capable of escaping the fibrin clot and initiating dissemination once they gained access to circulating hPg but that this process *in situ* was significantly delayed compared to that which we observed *in vitro*. We observed that, under the conditions in which GAS_AP53_–fibrin complexes are circulating in a 3D endothelial environment, fibrinolysis mediated by GAS was observed in the EMV after an incubation period of ~18 h. It should be noted here that endothelial cells contribute to hemostasis through their production of various proteins, among them, urokinase plasminogen activator (u-PA), tissue plasminogen activator (t-PA) and plasminogen activator inhibitor (PAI-1) (41). Although evidence suggests that HUVECs do not express abundant amounts of u-PA, they have been shown the productively secrete PAI-1 and t-PA (42). Production of t-PA did not appear to accelerate fibrinolytic activity in our model, possibly due to the presence of PAI-1. These studies reveal for the first time that a distinction between rates of fibrinolysis by SK-mediated hPg activation is dependent on the type of cellular system that is used to observe the process. Importantly, the 3D endothelial system that we have optimized for host–pathogen live imaging may provide investigators an important approach to more precisely model the process of pathogen dissemination and blood vessel spread that is characteristic of bacteremia and septic embolism.

## MATERIALS AND METHODS

### Bacterial cultures

The *Streptococcus pyogenes* isolate used in this study is an invasive clinical isolate from a patient with necrotizing fasciitis AP53 (GAS_AP53_) provided by Dr. Gunnar Lindahl (Lund, Sweden). This strain has enhanced invasive capabilities due to mutational inactivation of the sensor (S) component in the two-component control of virulence (cov) responder (R)/extracellular sensor (S) gene regulatory system, *covRS* (20). The natural mutations in this two-component system increase overall virulence with respect to the host (44). SK is one virulence factor under control of the CovRS system. An isogenic mutant (GAS_AP53_/ΔSK) was also used in this study and has been discussed previously (45). All GAS strains were grown overnight for 16–18 h in Difco TH broth (BD Bacto) or TH broth with 10% Bacto Yeast Extract (Gibco), referred to henceforth as THY, at 37°C prior to experimentation.

### Real-time live imaging

An inverted Nikon Eclipse Ti-E microscope fitted with an environmental chamber (set for 37°C with 5% CO2) at 20×–60× magnification was used to record in real-time the interactions of GAS_AP53_ and the fibrin clot. An iXon Ultra 897 electron-multiplying charge-coupled device (Andor) and a Neo sCMOS (Andor) were used to capture the images. Images were obtained in the FITC channel with a 480/30-nm excitation filter and 535/45-nm filter. Images were obtained for mCherry using the images that were analyzed and reconstructed by ImageJ/FIJI (46).

### Real-time live imaging of a fibrin clot infection model with GAS

Overnight GAS cultures in 4-mL TH broth were centrifuged, and the supernatant was removed. Moreover, 1× PBS (Gibco) was then added and vortexed with the centrifuged GAS_AP53_ pellet. After mixing, the GAS and PBS combination was used to dilute stock human fibrinogen (Enzyme Research Laboratories) to a concentration of 1 g/mL. This fibrinogen-GAS_AP53_ mixture was then placed into an optical imaging dish (Mattek). To convert fibrinogen to fibrin, thrombin (5 NIH units/mg, Enzyme Research Laboratories) was added to the imaging dish. The dish was set at 37°C with 5% CO2 for 1 h in an incubator. The samples were observed visually with microscopy to ensure the mixture and presence of bacteria and the formation of the fibrin clot prior to live imaging and addition of any other components to the experiment. To label the fibrin clot for microscopy, DyLight 488 NHS-Ester (Thermo Scientific), a fluorescent labeling reagent (50 µg/mL in PBS), was added to the clot. This labeled clot was allowed to incubate for 2 h in the dark at room temperature. After 2 h, two PBS washes were performed in order to remove excess reagent and bacteria not entrapped in the clot. The samples were then observed by microscopy to ensure that the fibrin clots contained enmeshed GAS_AP53_ bacteria. Then, TH media and hPg (7 µg/mL, provided by the W.M. Keck Center for Transgene Research) were added to the fibrin clot immediately preceding real-time imaging. Images were obtained every 10 min for 10 h. With certain experiments, hPg (at a concentration of 7 µg/mL) was added to the bacteria before the bacteria PBS solution was converted from fibrinogen to fibrin.

### Determination of GAS viability after entrapment in a fibrin clot

Fibrin clots containing GAS_AP53_ cells were formed following the aforementioned procedure for the GAS strains (GAS_AP53_ and GAS_AP53_/ΔSK). After clot formation occurred on the imaging device, the clot was placed into an incubator set at 37°C with 5% CO2. Triplicates were performed for each timepoint (4 and 8 h) and treatment. At each timepoint, the dish was removed from the incubator, and the clot was extracted from the dish into TH media. The clot was then mechanically disrupted in the TH solution using vortexing and pipette mixing of the suspension. This solution was then placed onto TH agar plates to quantify growth of GAS_AP53_ at timepoints and treatment conditions. These plates were placed at 37°C for 24 h. The plates were scored for colony growth. Statistical analyses were performed using Graphpad Prism.

### RNA isolation and RNA-Seq transcriptome analysis

GAS_AP53_ and GAS_AP53_/ΔSK cultures were grown overnight and entrapped within a clot employing the aforementioned method. After clot formation, hPg (7 µg/mL) was also added to clot. The clot was then incubated at 37°C at times of 4 and 8 h. Three replicates of each GAS strain at these times were processed for RNA sequencing. The clot was mechanically minced to be homogenous in TH media for RNA isolation. RNA isolation and analysis of the RNA-Seq were performed as previously described (47, 48). Briefly, a DNeasy Blood and Tissue Kit (Qiagen, Valencia, CA, USA) was used to extract and purify the RNA from GAS_AP53_ cells (Qiagen, Valencia, CA, USA). The sequencing library was constructed using an NEBNext rRNA Depeletion Kit (Bacteria) (New England Biolabs, Ipswich, MA, USA). From the kit, a NEBNext RNase H-based RNA depletion workflow was used to target removal of rRNA from Gram-positive and Gram-negative organisms. Quality control of the RNA preparation was checked using an Agilent 2100 Bioanalyzer System and Qubit RNA IQ Assay (Agilent Technologies, Santa Clara, CA, USA; Invitrogen, Waltham, MA, USA). This analysis provided an RNA integrity number (RIN) of >7.0. The Genomics and Bioinformatics Core Facility at the University of Notre Dame performed the RNA sequencing using an Illumina Miseq platform.

The sequencing reads were aligned to the GAS genome of AP53 (48) using Burrow Wheel Aligner (bwa; version 0.7.17) (49). The reads which were aligned to multiple *loci* were removed. The expression levels of genes were normalized using the method GeTMM (50). The differential expression of genes was performed between different treatments or time points and was calculated using edgeR (version 3.40.2) (51). The Benjamini–Hochberg multiple testing correction was applied to evaluate the FDR (52). PCAs were performed using R software (53).

### 3D-engineered endothelial system for live imaging

The 3D-engineered endothelial microfluidic device was generated from an original design as previously described (39). Human umbilical vein endothelial cells (HUVECs, Lonza) were cultured using endothelial growth medium (EGM-2, Lonza) with 10% FBS. Rat-tail Collagen I (Gibco; 2 mg/mL) was used to form an extracellular matrix hydrogel. After preparing the devices, HUVECs were briefly treated with 0.25% Trypsin/EDTA (Gibco) and counted using a hemacytometer. Cells were added to the wells of the device at a concentration of approximately 1.5 × 10^6^ cells/mL. Once HUVECs had adhered to the microvessel collagen surface, the devices were placed on a rocker, to generate oscillatory shear stress, and incubated at 37°C and 5% CO_2_ for 24–72 h prior to experimentation.

At the time of the experiment, overnight GAS_AP53_ cultures were centrifuged at 10,000 × *g* for 5 min prior to aspiration of supernatant, after which the media was replaced with sterile 1× PBS (pH 7.4). To prepare the GAS_AP53_ cells in a fibrin clot, the cells were first diluted to an OD_600nm_ of 0.4 by mixing with additional PBS and fibrinogen from human plasma with an Alexa Fluor 488 conjugate (0.1 mg/mL, Invitrogen). Lastly, thrombin (0.025 units/mL) was added to the clot mixture, and the mixture was placed in an incubator at 37°C for 1 h. After incubating the GAS–fibrin clot for 1 h, the mixture was centrifuged at 10,000× *g* for 5 min. The liquid fraction was aspirated, and THY was added to the sample pellet with an additional spin and wash step. The final pelleted fibrin clot containing GAS_AP53_ entrapped in the clot was resuspended by pipetting and scraping the side of the tubing with the pipet tip. A 60-µL volume of clot-THY suspension was subsequently added to each well of the EMV device for incorporation into the 3D endothelial system. Experiments that include hPg had hPg added directly after the GAS_AP53_–fibrin clots were added to the EMV wells at a concentration of 175 µg/mL. Live imaging of GAS–fibrin clots inside the 3D EMV device was conducted in a manner as previously described.

## SUPPLEMENTAL MATERIAL

Supplemental material spectrum.02607-24-s0001.docx Fig. S1; supplemental material captions.

Supplemental data spectrum.02607-24-s0002.xlsx RNA Sequencing Differential Expression Data WT and SK at 4 h and 8 h.

Movie S1 spectrum.02607-24-s0003.avi WT (AP53R+S-) GAS triggers fibrin clot dissolution at 6 h (DIC, 507 nm).

Movie S2 spectrum.02607-24-s0004.avi WT (AP53R+S-) GAS +hPg triggers fibrin clot dissolution at 4.5 h (DIC, 507nm).

Movie S3 spectrum.02607-24-s0005.avi SK (AP53R+S-) GAS +hPg does not trigger fibrin dissolution over 10 h (DIC).

Movie S4 spectrum.02607-24-s0006.avi SK (AP53R+S-) GAS +hPg does not trigger fibrin dissolution over 10 h (507 nm).

Movie S5 spectrum.02607-24-s0007.avi WT (AP53R+S-) GAS does not show significant growth over 10 h trapped in fibrin.

Movie S6 spectrum.02607-24-s0008.mov WT (AP53R+S-/mCherry) GAS infection of a 3-D EMV device containing fibrin clots and HUVECs.

Movie S7 spectrum.02607-24-s0009.mov Fibrin-trapped WT (AP53R+S-/mCherry) GAS infection of an EMV device, hPg is added simultaneously.

Movie S8 spectrum.02607-24-s0010.avi Fibrin-trapped WT (AP53R+S-/mCherry) GAS infection of an EMV device, hPg is pre-incubated with GAS.

ASM does not own the copyrights to Supplemental Material that may be linked to, or accessed through, an article. The authors have granted ASM a non-exclusive, world-wide license to publish the Supplemental Material files. Please contact the corresponding author directly for reuse.
