# Supplementary material for: Group a Streptococcus remains viable inside fibrin clots and gains access to human plasminogen for subsequent fibrinolysis and dissemination
Source: Microbiol Spectr. 2025 Jan 13;13(2):e02607-24. doi: 10.1128/spectrum.02607-24 (PMC11792473; doi:10.1128/spectrum.02607-24)

**Supporting Information**

**S1 Fig. WT (AP53R^+^S^-^) GAS does not show significant growth over 10 h while trapped in fibrin without hPg. DIC images of live imaging time course shows no appreciable growth of GAS bacteria while enmeshed in fibrin clots. 0, 4, 8, 10 h images shown.**

**S1 Movie. WT (AP53R^+^S^-^) GAS triggers fibrin clot dissolution at 6 h (DIC, 507 nm). DIC channel of sustained live imaging time course movie demonstrating rapid fibrinolytic event by WT GAS after 6h (left). Live imaging time course movie of fluorescently labeled fibrin clot dissolution by WT GAS (right). hPg is added exogenously to GAS-enmeshed clots prior to live imaging.**

**S2 Movie. WT (AP53R^+^S^-^) GAS +hPg triggers fibrin clot dissolution at 4.5 h (DIC, 507nm). DIC channel of sustained live imaging time course movie demonstrating rapid fibrinolytic event by WT GAS after 4.5 h (left). Live imaging time course movie of fluorescently labeled fibrin clot dissolution by WT GAS (right). hPg is pre-incubated with GAS prior to placement in enmeshed fibrin clots.**

**S3 Movie. ΔSK (AP53R^+^S^-^) GAS +hPg does not trigger fibrin dissolution over 10 h (DIC). DIC channel of sustained live imaging time course movie over 10h demonstrating that GAS bacteria lacking SK cannot initiate fibrinolysis and remain enmeshed in the fibrin clot without growth.**

**S4 Movie.** **ΔSK (AP53R^+^S^-^) GAS +hPg does not trigger fibrin dissolution over 10 h (507 nm).**

**Sustained live imaging time course movie over 10h demonstrating that bacteria lacking SK cannot initiate fibrinolysis and remain enmeshed in the fibrin clot without growth. Fluorescently labeled fibrin is imaged throughout the course of the experiment.**

**S5 Movie. WT (AP53R^+^S^-^) GAS does not show significant growth over 10 h trapped in fibrin. DIC live imaging time course shows no appreciable growth of GAS bacteria while enmeshed in fibrin clots in the absence of hPg.**

**S6 Movie. WT (AP53R^+^S^-^/mCherry) GAS infection of a 3-D EMV device containing fibrin clots and HUVECs.** Sustained live imaging with mCherry expressing wt GAS bacteria and fluorescently labeled fibrin, showing rapid onset of fibrinolysis after 6h when hPg and bacteria are introduced into the 3-D EMV device containing fibrin clots.

**S7 Movie. Fibrin-trapped WT (AP53R^+^S^-^/mCherry) GAS infection of an EMV device, hPg is added simultaneously.** Sustained live imaging with wt GAS bacteria and fluorescently labeled fibrin, showing onset of fibrinolysis after 18h when hPg is added to the device when bacteria are enmeshed in fibrin clots prior to addition of hPg.

**S8 Movie. Fibrin-trapped WT (AP53R^+^S^-^/mCherry) GAS infection of an EMV device, hPg is** **pre-incubated with GAS.** Sustained live imaging with wt GAS bacteria and fluorescently labeled fibrin, showing onset of fibrinolysis after 18h when hPg is preincubated with bacteria prior to being enmeshed in fibrin clot.

**S1 Data. RNA Sequencing Differential Expression Data WT and ΔSK at 4 h and 8 h.**

RNA-seq analyses of WT-GAS_AP53_and GAS_AP53_/ΔSK compared along with 4 h and 8 h time points of each GAS strain. Complete profile of all gene expression changes is listed in table form.

**Figure S1**


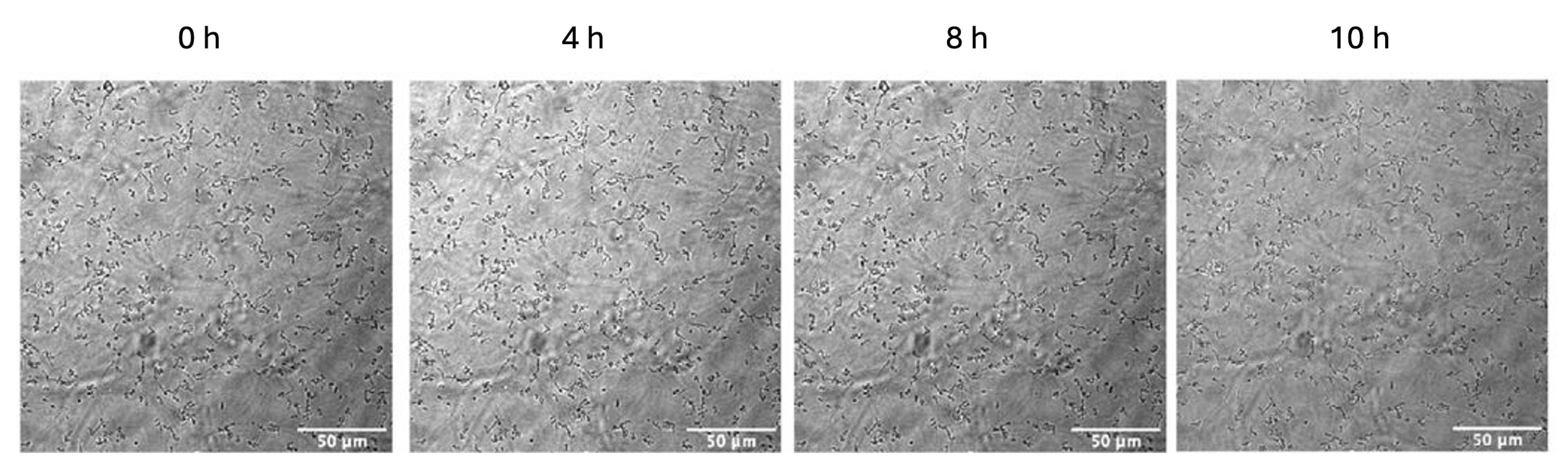

Supplement: Supplemental material — Fig. S1; supplemental material captions. [file spectrum.02607-24-s0001.docx]
